# A novel *Klebsiella pneumoniae* diguanylate cyclase contributes to intestinal cell adhesion, biofilm formation, iron utilization, and *in vivo* virulence by gastrointestinal infection

**DOI:** 10.1080/21505594.2025.2544882

**Published:** 2025-08-06

**Authors:** Chun-Ru Hsu, Ya-Ling Huang, Pang-Hung Hsu, Chen-Hsiu Huang, Yu-Chieh Huang, Ming-Ti Kao, You-Wei Ye

**Affiliations:** a Department of Bioscience and Biotechnology, National Taiwan Ocean University, Keelung, Taiwan; b Department of Laboratory Medicine, E-Da Hospital, I-Shou University, Kaohsiung, Taiwan; c Department of Medical Laboratory Science, College of Medical Science and Technology, I-Shou University, Kaohsiung, Taiwan; d Center of Excellence for the Oceans, National Taiwan Ocean University, Keelung, Taiwan

**Keywords:** Klebsiella pneumoniae, gastrointestinal interaction, diguanylate cyclase, cyclic di-GMP, biofilm

## Abstract

*Klebsiella pneumoniae* is responsible for various infections such as bacteremia, urinary tract infections, pneumonia, and liver abscesses. Multidrug-resistant *K. pneumoniae* infections pose a critical public health threat, often associated with high mortality rates. The emergence of hypervirulent *K. pneumoniae* has also raised global health concerns due to its invasive disease potential. Clinical studies suggest intestinal colonization by *K. pneumoniae* as a risk factor for subsequent infections, but the underlying mechanisms remain unclear. Cyclic di-GMP (c-di-GMP), a bacterial signaling molecule synthesized by diguanylate cyclases (DGCs), controls various cellular processes and is absent in higher organisms, making it an attractive target for antimicrobial development. In this study, we identified a novel DGC, designated as DgcG, in *K. pneumoniae*, which plays a pivotal role in gastrointestinal colonization and pathogenesis. Using genetic deletion and complementation analyses in a bacteremia and liver abscesses-inducing strain Ca0437, we observed that DgcG promoted intestinal adherence, biofilm formation, iron utilization, and *in vivo* virulence. RT-qPCR showed that DgcG regulated genes involved in type 3 fimbrial expression and iron transport. In a gastrointestinal infection model of female BALB/cByl mice, *dgcG* deletion significantly reduced host mortality and bacterial load in the liver, spleen, and intestines, underscoring its role in enhancing bacterial survival and dissemination. Additionally, *dgcG* gene was found highly conserved and prevalent among diverse *K. pneumoniae* isolates. These findings provide new insights into c-di-GMP-mediated virulence regulation in *K. pneumoniae* and highlight DgcG as a potential therapeutic target for controlling *K. pneumoniae* infections, especially amidst the growing global antimicrobial resistance crisis.

## Introduction

*Klebsiella pneumoniae* is a significant human pathogen associated with a range of diseases. The World Health Organization (WHO) has designated *K. pneumoniae* as a critical priority among healthcare-associated pathogens [1]. It is a leading cause of hospital-acquired infections globally, including urinary tract infections (UTIs), pneumonia, wound infections, and septicemia, particularly in immunocompromised individuals [2–4]. *K. pneumoniae* is one of the “ESKAPE” pathogens, a group of six major bacterial species driving the global antimicrobial resistance crisis in healthcare settings [5]. According to 2019 global burden studies on bacterial antimicrobial resistance, *K. pneumoniae* ranks among the leading pathogens responsible for drug-resistant-related fatalities [6]. A distinct infection, known as community-acquired pyogenic liver abscess (PLA), caused by *K. pneumoniae* has emerged in Taiwan and other regions [7–10]. These severe, invasive infections are primarily attributed to hypervirulent *K. pneumoniae* (hvKp), which expresses acquired virulence factors. The emergence and global spread of multidrug-resistant (MDR) and hypervirulent clones have made *K. pneumoniae* a serious clinical and public health concern [11,12].

Cyclic di-GMP (c-di-GMP) is a global second messenger in bacteria, regulating diverse cellular processes [13]. Diguanylate cyclases (DGCs) and phosphodiesterases (PDEs) have opposing roles in c-di-GMP synthesis and degradation. Synthesis is mediated by DGCs with GGDEF domains (characterized by a conserved GG(D/E)EF motif), while degradation occurs via PDEs containing either EAL domains (with a conserved E(A/V)L motif) or HD-GYP domains [13]. The c-di-GMP molecule links environmental or intracellular signals to changes in bacterial motility, development, biofilm formation, exopolysaccharide synthesis, and virulence gene expression [13]. As c-di-GMP signaling is absent in higher eukaryotes, it presents an attractive target for antimicrobial development [14–17]. Small-molecule DGC inhibitors have been screened to create anti-biofilm agents against pathogens such as *Pseudomonas aeruginosa* [15], *Acinetobacter baumannii* [15], and *Vibrio cholerae* [16]. Strategies targeting c-di-GMP signaling or essential DGCs are gaining attention as potential therapies for antibiotic-resistant bacteria [17].

Many bacterial genomes encode numerous putative *dgc* and *pde* genes, suggesting complex, species-specific c-di-GMP regulation [18,19]. C-di-GMP interacts with various effectors and receptors, modulating downstream targets through intricate regulatory mechanisms [20]. In *K. pneumoniae*, c-di-GMP regulates biofilm formation, type 3 fimbriae, capsular polysaccharide production, and oxidative stress responses [21–23]. Despite the diversity of putative DGCs and PDEs in its genome [24], their roles and regulatory networks in pathogenesis remain largely uncharacterized.

The intestine serves as a major reservoir for *K. pneumoniae* in humans. Clinical studies suggest infections such as liver abscess and bacteremia may be preceded by gastrointestinal (GI) colonization [25–28]. Cohort studies report that intestinal colonization is a risk factor for subsequent infection [25,26]. Capsular polysaccharides and type 1 and 3 fimbriae contribute to attachment and colonization [29]. However, the molecular mechanisms and regulation of *K. pneumoniae*-GI interactions remain unclear.

To investigate these interactions, our previous work screened a transposon mutant library and identified a mutant with disruption in *kpn02265*, a gene essential for adherence to Caco-2 intestinal epithelial cells [30]. Gene *kpn02265* encodes a putative DGC containing a GGDEF domain. In this study, we demonstrate that *kpn02265* encodes a functional DGC, designated DgcG (diguanylate cyclase for gastrointestinal interaction), which plays a crucial role in host interaction. Through genetic deletion and complementation experiments, we show that DgcG contributes to adherence, type 3 fimbrial expression, biofilm formation, and iron utilization. A mouse model of gastrointestinal infection revealed that DgcG enhances bacterial survival and *in vivo* virulence. Moreover, the *dgcG* gene is universally conserved among clinical *K. pneumoniae* isolates.

## Materials and methods

### Bacterial strains and genetic manipulation

*K. pneumoniae* Ca0437 strain is a clinical isolate with K2 capsular type originally obtained from the blood of a patient with septicemia [31]. Ca0437 exhibited a great ability of adhesion to and invasion into intestinal epithelial cells [32], and induced liver abscess through gastrointestinal infection in a murine model [30]. Ca0437 wild type and *dgcG* deletion mutant (Δ*dgcG*) was gifted from Professor Jin-Town Wang from the National Taiwan University (NTU). Ca0437 isogenic mutant with unmarked deletion of gene was generated using the temperature-sensitive pKO3-Km plasmid in a method as described previously [30]. Genetic complementation of *dgcG* in Δ*dgcG* strain was constructed by chromosomal integration of a single copy of *dgcG* using the pKO3-Km vector according to a previously reported method [30]. Gene deletion and complementation were confirmed by PCR amplification and sequencing. *K. pneumoniae* isolates for prevalence analysis were collected from the E-Da Hospital. The bacterial strains used in this study are listed in supplementary Table S1. The sequence types (STs) and capsule locus types (KL types) of 70 gut isolates, determined by whole genome sequencing analysis (unpublished data), are provided in Dataset 1 of supplementary files.

### Determination of growth kinetics

Bacterial single colonies were inoculated into Luria-Bertani (LB) broth and grown overnight at 37°C with shaking at 200 rpm. Overnight cultures were diluted in fresh LB broth to an initial optical density at 600 nm (absorbance value A₆₀₀) of 0.05. Growth was monitored at 37°C by measuring A₆₀₀ at each time point: 0, 0.5, 1, 2, 3, 4, 5, 6, 7, 8, 24 h.

### Cell adherence assays

Four cell lines were tested: Caco-2 (intestinal epithelial), T24 (bladder epithelial), ARPE-19 (retinal epithelial), and RAW 264.7 (murine macrophages). T24, ARPE-19, and RAW 264.7 cells were purchased from the Bioresource Collection and Research Center (BRCR), Taiwan (https://www.bcrc.firdi.org.tw/). Caco-2 cells were gifted from Professor Jin-Town Wang (NTU). Cells were cultured in appropriate media supplemented with 10% fetal bovine serum (FBS). Bacteria at mid-log phase were suspended in serum-free media and added to cells at a multiplicity of infection (MOI) of 50. After centrifugation, cells were incubated, washed with PBS, and treated with Triton X-100 to release adhered bacteria. Quantification was performed by plating and counting colony forming units (CFUs). The adherence rate was calculated as the proportion of the inoculum adhering to cells. Three independent experiments were conducted. Statistical significance was assessed using analysis of variance followed by Bonferroni multiple comparisons test. Details are described in the Supplementary Methods.

### Prevalence and sequences analysis of dgcG in K. pneumoniae

Ca0437 *dgcG* was sequenced based on Sanger sequencing by commercial service (Genomics BioSci & Tech. Co. Ltd., Taiwan). Protein domain was predicted using SMART program (http://smart.embl-heidelberg.de/). Amino acid sequence alignments, similarities, and phylogeny were analyzed using Clustal Omega multiple sequence alignment program (https://www.ebi.ac.uk/jdispatcher/msa/clustalo). The tBLASTn program was used to identify DgcG in *K. pneumonia* genomes. The publicly available *K. pneumonia* genomes were obtained from the NCBI database (https://www.ncbi.nlm.nih.gov/datasets/genome/). The list of 636 *K. pneumonia* genomes for DgcG comparison was in Dataset 1 of supplementary files. *K. pneumonia* STs and KL types were determined using Kleborate program (https://github.com/klebgenomics/Kleborate).

### Expression of dgcG and c-di-GMP quantification

To verify the function of Ca0437 *dgcG*, the gene was cloned into the pJET1.2 vector and expressed in *Escherichia coli* BL21 (DE3). Expression was induced with 0.4 mM isopropyl β-D-1-thiogalactopyranoside (IPTG) for four hours at 37°C. Cellular c-di-GMP was extracted and quantified using high-performance liquid chromatography (HPLC), as detailed in the Supplementary Methods.

### Biofilm assays and scanning electron microscopy (SEM)

*K. pneumoniae* biofilm was quantified using the crystal violet (CV)-based biofilm assays [33]. In brief, *K. pneumoniae* overnight culture was diluted to A_600_ = 0.05 in LB and incubated statically at 37°C for 24 h to allow biofilm formation. Bacterial growth was measured at A_600_ to estimate total cell biomass. The culture medium was carefully removed and biofilms were fixed with methanol and stained with 0.1% of CV for 15 min, followed by wash with sterile distilled water three times to remove excess dye and allowed to dry at room temperature. The CV was dissolved in 200 μL of 95% ethanol and the optical density at 570 nm was measured. The amount of biofilm formed was determined from the A_570_/A_600_ ratio, to compensate for variations due to differences in bacterial growth [34]. Three independent experiments were conducted. Statistical significance was assessed using analysis of variance followed by Bonferroni multiple comparisons test.

To validate potential differences of biofilm, SEM was used to observe the structures and morphology of *K. pneumoniae* biofilm according to previously described methods [35]. *K. pneumoniae* overnight culture was diluted to A_600_ = 0.05 in LB and seeded on sterile coverslips in the wells of a 6-well plate, followed by static incubation at 37°C for 24 h. After removal of culture, biofilms were washed with PBS, fixed with 2.5% glutaraldehyde at 4°C for 4 h, and further subjected to a gradient series of ethanol (30, 50, 70, 90, and 100% v/v), each for 10 min. The samples were dried for 2 h and observed using SEM (Hitachi, S-3400N).

### Iron utilization and serum resistance assays

For iron utilization analysis [36], bacteria were grown in iron-free chemically defined DIS medium and then tested with or without Fe^3+^ (500 µM FeCl_3_) or Fe^2+^ (500 µM FeSO_4_). *K. pneumoniae* was grown overnight on LB agar at 37°C. A single bacterial colony in 5 mL iron-free DIS medium was equilibrated to an optical density at 600 nm (absorbance value A_600_) of 0.1 for iron-starvation at 37°C for 24 h. Iron-starved bacteria were diluted with DIS medium appropriately to match cultures to 1 × 10^7^ CFU/mL. Iron-starved bacteria (4 µL) were added to the wells of 96-well polystyrene microplate containing 100 µL of DIS supplemented with 0 or 500 µM FeCl_3_ (for Fe^3+^)/FeSO_4_ (for Fe^2+^), in triplicate. The plate was incubated at 37°C, and A_600_ was measured at various time points to assess growth.

Serum resistance was determined by incubating bacteria with human serum for two hours, followed by CFU enumeration. A *K. pneumoniae* inoculum of 2.5 × 10^4^ CFU in 25 μL were mixed with 75 μL human serum from the healthy volunteers. The mixtures were incubated at 37 °C for 2 h. After serial dilution and plating, the numbers of CFU were determined. The bacterial survival ratio was expressed as recovered CFUs/inoculum CFUs. The survival ratios ≥1 indicated serum resistance [37].

### RNA-sequencing (RNA-seq) and real-time reverse-transcription PCR (RT-qPCR)

The transcriptomes of *K. pneumoniae* were analyzed using RNA-seq conducted by a commercial service (AllBio Science., Inc, Taiwan). Total RNA was extracted from log-phase bacteria, ribosomal RNA was removed, and libraries were prepared for Illumina HiSeq sequencing. Differential expression analysis was performed using DESeq2, and enriched pathways were identified through Gene Ontology (GO) and KEGG analysis. For real-time reverse-transcription PCR (RT-qPCR), RNA was extracted and treated with DNase I. Reverse transcription and amplification were performed using SYBR Green. For each gene, the calculated threshold cycle (Ct) was normalized to the Ct of the 23*S* ribosomal RNA gene. The relative RNA expression was calculated based on the ΔΔCt value. RT-qPCR primers used in this study are listed in supplementary Table S2.

### Mouse experiments

All animal experiments were approved by the Institutional Animal Care and Use Committee of National Taiwan Ocean University (IACUC-109060). The study has adhered to the ARRIVE guidelines. Mouse survival, bacterial load, and *in vivo* competition assays were performed in a murine gastrointestinal infection model using five-week-old female BALB/cByl mice purchased from the National Laboratory Animal Center, Taiwan.

For survival analysis, mice (8 per group) were intragastrically (IG) administered 6 × 10^6^ CFUs of mid-log-phase *K. pneumoniae* in 100 μL of saline solution and monitored daily for 30 days. Mouse survival was analyzed by means of Kaplan-Meier analysis using a log-rank test with Bonferroni correction.

Bacterial load experiments involved IG administration of 1 × 10^7^ CFUs in 100 μL of saline solution to mice (10 per group), followed by organ harvesting (liver, spleen, and colorectum) at 72 h post-infection. Recovered CFUs from organ homogenates were standardized to 0.1 g wet organ weight. Statistical significance was assessed using analysis of variance followed by Bonferroni multiple comparisons test.

For *in vivo* competition assays [35], the test strain (Ca0437 WT, Δ*dgcG* or Δ*dgcG::dgcG*) was mixed with the fully virulent isogenic *lacZ* promoter deletion mutant (Ca0437Δp*lacZ*) at a 1:1 ratio. A per-mouse dose of 1 × 10^8^ CFU in 100 μL of saline solution was inoculated IG into each BALB/cByl mouse (8 per group). At 24 h post-inoculation, the liver and spleen were removed and homogenized in 1× PBS; bacteria were recovered by plating appropriate dilutions on LB plates containing 1 mmol/ml IPTG and 50 µg/ml X-Gal. The number of LacZ-positive (blue) and LacZ-negative (white) colonies were counted. The competitive index (CI) was defined as (output_test strain_/output_Δp*lacZ*_)/(input_test strain_/input_Δp*lacZ*_); the resulting ratio was interpreted as the *in vivo* colonization ability [38]. Statistical significance was assessed using analysis of variance followed by Bonferroni multiple comparisons test.

### Statistical analyses

Data are presented as means with standard errors of the mean (SEM). Statistical significance was assessed using analysis of variance followed by a post hoc multiple comparisons test performed with Prism 5 software (GraphPad). Differences were considered significant at *p* < 0.05.

## Results

### Identification of a novel *K. pneumoniae* DGC promoting adherence to intestinal cells

**Figure 1.**
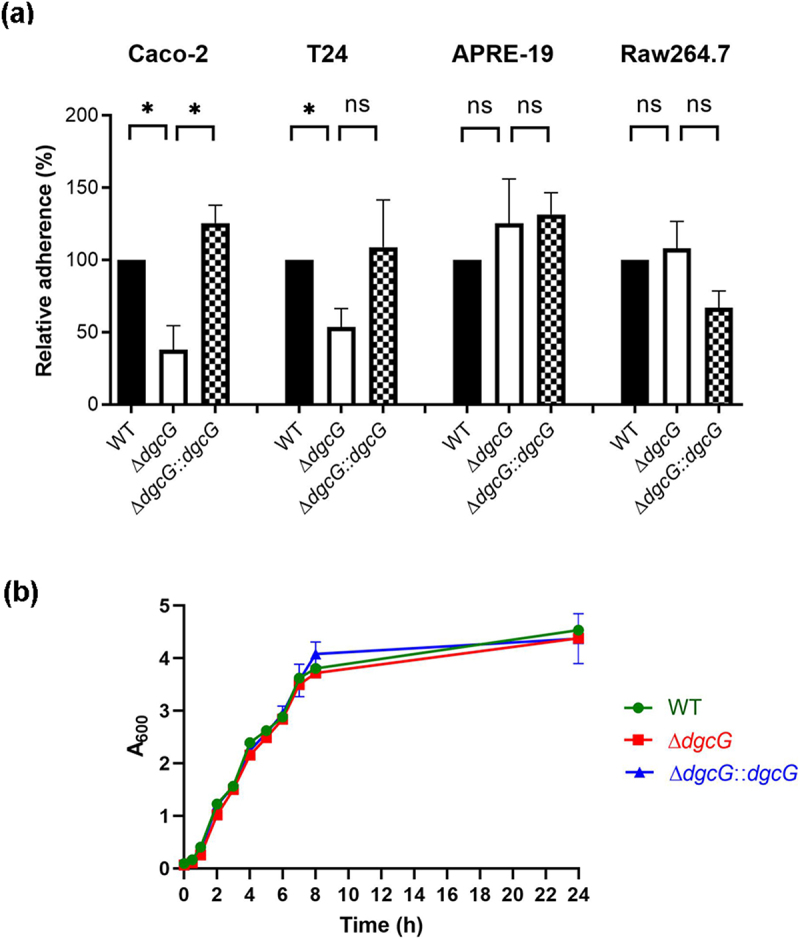
*K. pneumoniae* DgcG promoted adherence to human intestinal cells. (a) Cell adherence assays. Adherence of *K. pneumoniae* Ca0437 wild type (WT), *dgcG* deletion mutant (Δ*dgcG*), and complementation strain (Δ*dgcG::dgcG*) to different types of cells were analysed: caco-2 intestinal epithelial cells, T24 urinary bladder epithelial cells, ARPE-19 retinal pigment epithelial cells and RAW264.7 macrophages. Relative adherence rates were normalized to that of the WT parent strain (defined as 100%). Bars represent the mean and standard error of the mean from ≥ 3 independent experiments. *, *p* < 0.05; ns, not significant; analysis of variance followed by Bonferroni multiple comparisons test. (b) Growth kinetics. Separate overnight cultures of each *K. pneumoniae* strain were inoculated into fresh LB broth and grown at 37°C. The growth of bacteria was monitored by measuring absorbance at 600 nm (A_600_).

Previously, we identified *dgcG* (*kpn02265*) through transposon-insertion mutagenesis screening of a bacteraemia-inducing clinical strain *K. pneumoniae* Ca0437 [30]. This strain exhibited a great ability of intestinal cell adhesion [32] and induced liver abscess by gastrointestinal infection in a murine model [30]. To verify the *dgcG* effects on cell interactions, we further analysed the in-frame unmarked gene deletion mutant (Δ*dgcG*) and chromosomal gene complementation (Δ*dgcG::dgcG*) strains for cell adherence (Figure 1a). Since *K. pneumoniae* can cause infections in various tissues [2–4,7–10], we evaluated its adherence to different cell types, including intestinal epithelial cells, urinary bladder epithelial cells, retinal pigment epithelial cells, and macrophages. Cell adherence assays showed that deletion of *dgcG* led to a significant reduction in adherence to Caco-2 intestinal epithelial cells (62% reduction compared to the wild type) and T24 urinary bladder epithelial cells (42% reduction compared to the wild type). No significant impact was observed on adherence to APRE-19 cells or RAW 264.7 macrophages. Genetic complementation significantly restored the defect in adherence to Caco-2 cells, resulting in an 88% increase compared to the mutant. It also rescued adherence to T24 bladder epithelial cells, with a 55% increase relative to the mutant. These data confirmed that *dgcG* was important for *K. pneumoniae* adherence to intestinal cells. Deletion of *dgcG* did not cause changes in bacterial growth in the nutrient broth (Figure 1b).

**Figure 2.**
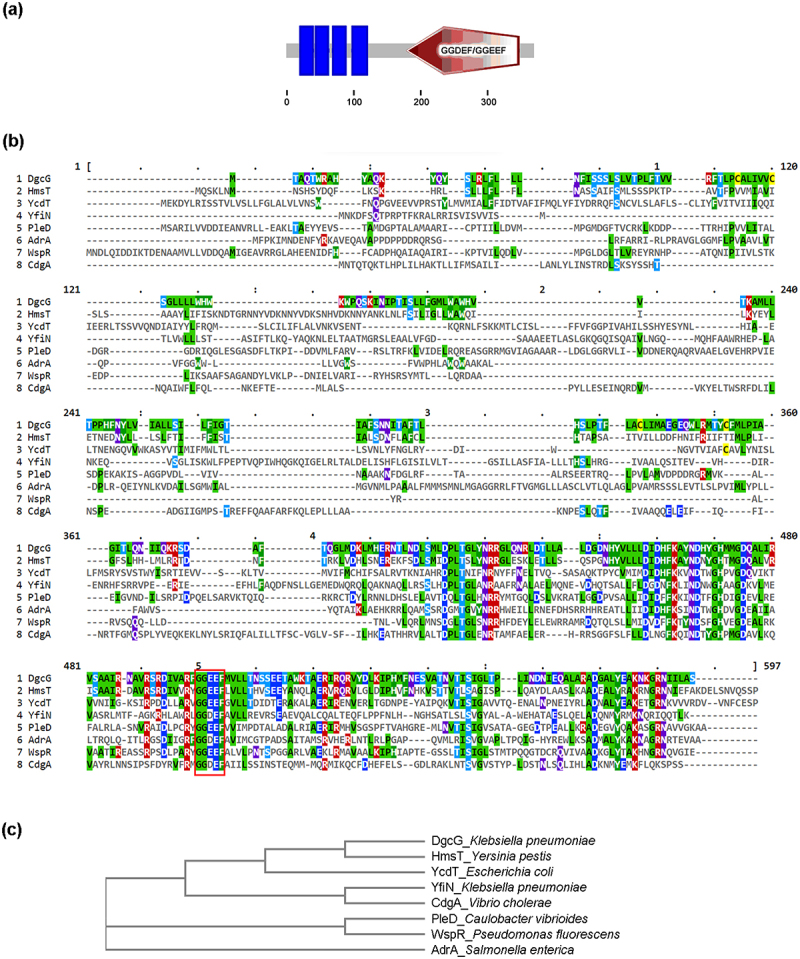
Domain prediction and comparison of *K. pneumoniae* Ca0437 DgcG to other well-characterized DGCs.(a) Domain prediction of *K. pneumoniae* DgcG by SMART program (http://smart.Embl-heidelberg.De/). DgcG contained a predicted GGDEF/GGEEF domain and four transmembrane (TM) domains (indicated in blue bars). (b) Amino acid sequence alignments of *K. pneumoniae* DgcG and other well-characterized DGCs, by Clustal Omega multiple sequence alignment program and MView (https://www.ebi.ac.uk/jdispatcher/msa/clustalo). The red box indicated the GGDEF/GGEEF domain. Accession No.: AAD25088.1 for HmsT_*Yersinia pestis*, NP_415544.1 for YcdT (DgcT)_*Escherichia coli*, WP_002914116.1 for YfiN (DgcN)_*Klebsiella pneumoniae*, AAA87378.1 for PleD_*Caulobacter vibrioides*, ELX61125.1 for AdrA_*Salmonella enterica*, AAL71852.1 for WspR_*Pseudomonas fluorescens*, ACP10914.1 for CdgA_*Vibrio cholera*. (c) Phylogenetic tree of *K. pneumoniae* DgcG and other DGCs.

Sequence-based functional prediction of the *K. pneumoniae* Ca0437 *dgcG* gene indicated that it encoded a putative diguanylate cyclase with a GGDEF/GGEEF domain and four transmembrane regions (Figure 2a). Ca0437 *dgcG* sequences showed 100% identical to KPN2242_02265 (accession number AEJ96373.1) in *K. pneumoniae* KCTC2242 complete genome from NCBI database. Comparative analysis revealed conservation of Ca0437 DgcG and other well-characterized DGCs from various bacterial species (Figure 2b). Phylogenetic analysis demonstrated that *K. pneumoniae* DgcG shares the closest relationship with *Yersinia pestis* HmsT (Figure 2c). Sequence similarities of *K. pneumoniae* DgcG to these well-characterized DGCs are shown in Table 1. Amino acid similarities ranged from ~21% to 46.84%. We also compared *K. pneumoniae* DgcG to the designated DGCs in *E. coli* since systematic nomenclature of DGCs has been established for *E. coli* [39]. The amino acid similarities between DgcG and14 *E. coli* DGCs were all below 30% (Table 1), suggesting that DgcG is distinct from these known *E. coli* DGCs.

**Table 1.** Comparison of *K. pneumoniae* DgcG to other well-characterized DGCs and known *E. coli* DGCs.

DGCs	Accession number	Other name	Amino acid number	Amino acid sequence similarities to DgcG (%)^*a*^
**Experimentally characterized**				
HmsT [*Yersinia pestis*]	AAD25088.1		390 aa	46.84
PleD [*Caulobacter vibrioides*]	AAA87378.1		454 aa	26.73
AdrA [*Salmonella enterica*]	ELX61125.1	DgcC [*E. coli*]; YaiC [*E. coli*]	370 aa	26.28
WspR [*Pseudomonas fluorescens*]	AAL71852.1		333 aa	30.47
CdgA [*Vibrio cholerae*]	ACP10914.1		366 aa	23.55
YcdT [*E. coli*]	NP_415544.1	DgcT [*E. coli*]	452 aa	25.81
YfiN [Klebsiella pneumoniae]	WP_002914116.1	DgcN [*E. coli*]; TpbB [Pseudomonas aeruginosa]	407 aa	21.30
**Other DGCs in *E. coli***				
DgcE [*E. coli*]	NP_416571.1	YegE [*E. coli*]	1105 aa	26.89
DgcF [*E. coli*]	NP_416039.1	YneF [*E. coli*]	315 aa	26.55
DgcI [*E. coli*]	NP_415355.1	YliF [*E. coli*]	442 aa	23.36
DgcJ [*E. coli*]	NP_416300.2	YeaJ [*E. coli*]	496 aa	25.00
DgcM [*E. coli*]	NP_415857.2	YdaM [*E. coli*]	410 aa	28.31
DgcO [*E. coli*]	NP_416007.3	DosC [*E. coli*]; YddV [*E. coli*]	460 aa	29.43
DgcP [*E. coli*]	NP_416308.4	YeaP [*E. coli*]	341 aa	25.16
DgcQ [*E. coli*]	NP_416465.2	YedQ [*E. coli*]	564 aa	24.40
DgcX [*E. coli*]	CAU96679.1		443 aa	26.25
DgcY [*E. coli*]	ACB15888.1		349 aa	29.79
DgcZ [*E. coli*]	NP_416052.1	YdeH [*E. coli*]	296 aa	26.83
**Others**				
DgcA [*Treponema denticola*]	Q73RG3		371aa	27.48
DgcB [*Bdellovibrio bacteriovorus*]	Q6MPU8		320 aa	30.03

*a*. Compared with *K. pneumoniae* Ca0437 (348 aa, accession number PQ435165). Sequence alignment and similarity was determined using Clustal Omega (https://www.ebi.ac.uk/Tools/msa/clustalo/).

To validate the diguanylate cyclase function, *K. pneumoniae* Ca0437 *dgcG* was cloned and expressed in *E. coli* BL21 (DE3). Intracellular c-di-GMP levels were quantified by HPLC following IPTG-induced expression of *dgcG*, which led to a significant increase in c-di-GMP levels (supplementary Table S3). This confirmed the diguanylate cyclase activity of DgcG to synthesize c-di-GMP molecules.

### K. pneumoniae DgcG contributes to biofilm formation and iron utilization

**Figure 3.**
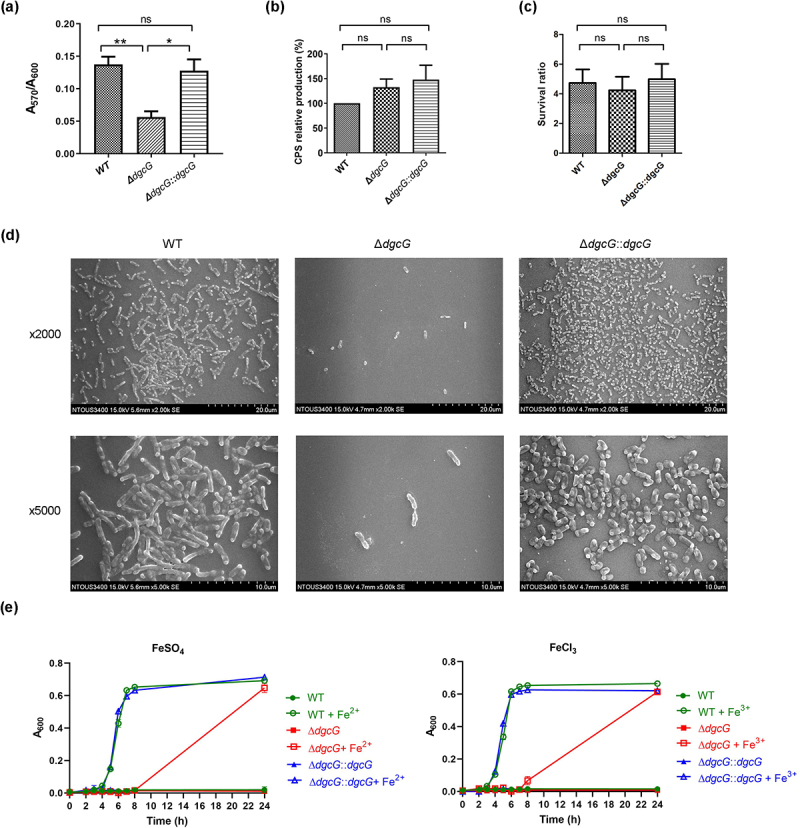
*K. pneumoniae* DgcG contributed to biofilm formation and iron utilization. Virulence-associated traits and growth of *K. pneumoniae* Ca0437 wild-type (WT), *dgcG*-deletion mutant (Δ*dgcG*), and complementation strain (Δ*dgcG::dgcG*) were determined and compared. (a) Quantification of biofilm formation. Biofilm levels were measured by crystal violet-based biofilm assays (see methods) and quantified as the A_570_/A_600_ ratio to normalize for differences in bacterial growth. Each bar indicates the mean ± SEM from three independent experiments. (b) Capsular polysaccharide (CPS) production. The levels of extracted CPS from indicated strains were determined and relative production were normalized to WT (defined as 100%). Each bar indicates the mean±SEM from three independent experiments. (c) Serum resistance. Indicated strains were treated with human serum (75%) at 37°C for 2 hours. The numbers of recovered CFUs were determined, and the survival ratio was expressed as recovered CFUs/inoculum CFUs. (d) Scanning electron microscopy (SEM) images of biofilm formation. Equivalent amount of *K. pneumoniae* strains were grown on sterile coverslips for 24 h. After removal of culture, biofilms were washed with PBS and fixed with 2.5% glutaraldehyde for SEM observation at 2,000x (upper graph) and 5,000x (lower graph). (e) Iron utilization with Fe^3+^ (500 µM FeCl_3_, left graph) or Fe^2+^ (500 µM FeSO_4_, right graph). Bacterial strains were starved for iron for 24 hours and then shifted to DIS cultures supplemented with iron (+ Fe^3+^ or Fe^2+^) or without iron. Growth was assessed by monitoring the increase in culture absorbance at 600 nm (A_600_) at the time points indicated. Data in (a), (b) and (c) are presented as the mean±SEM from at least three independent experiments. Throughout figure, *, *p* < 0.05; **, *p* < 0.01; ns, not significant; analysis of variance followed by Bonferroni multiple comparisons test.

We examined whether *dgcG* influenced virulence-associated traits of *K. pneumoniae*, including biofilm formation, capsular polysaccharide (CPS) production, serum resistance, and iron utilization (Figure 3a–d). Interestingly, we observed that *dgcG* contributed to *K. pneumoniae* biofilm formation and iron utilization. The crystal violet-based biofilm assays (Figure 3a) showed that deletion of *dgcG* caused the decrease of biofilm formation, and the defects were rescued by genetic complementation. The differences of biofilm formation were observed using scanning electron microscope (SEM) (Figure 3d). Consistent with the results of the crystal violet assay, the Δ*dgcG* mutant exhibited a lack of biofilm formation compared to the wild type (WT), and this defect was restored by genetic complementation.

Iron utilization assays (Figure 3e) showed that the Δ*dgcG* mutant was deficient in iron utilization during the 0–8 h period, including the use of Fe^3 +^ (left graph, FeCl₃) and Fe^2 +^ (right graph, FeSO₄). While iron-starved WT bacteria failed to grow in iron-depleted medium, their growth was restored in the presence of either Fe^3+^ or Fe^2+^. In contrast, the iron-starved Δ*dgcG* mutant was unable to resume growth with either form of iron by 8 h. This defect in iron utilization was rescued by genetic complementation. By 24 h, no differences in growth were observed among the WT, Δ*dgcG*, and Δ*dgcG::dgcG* strains. Therefore, deletion of *dgcG* caused delayed kinetics and appeared to reduce the efficiency of iron utilization, particularly during the exponential growth phase.

Production of CPS (Figure 3b) and serum resistance (Figure 3c) were not significantly influenced by deletion of *dgcG*. In addition, the string tests and low-speed centrifugation assays to analyze *K. pneumoniae* mucoviscosities both showed no significant differences between WT, Δ*dgcG*, and Δ*dgcG::dgcG* strains (data not shown), in agreement with observation that *K. pneumoniae* DgcG might not regulate CPS biosynthesis.

### *K. pneumoniae* DgcG regulates expression of type 3 fimbriae and iron transport-related genes

**Figure 4.**
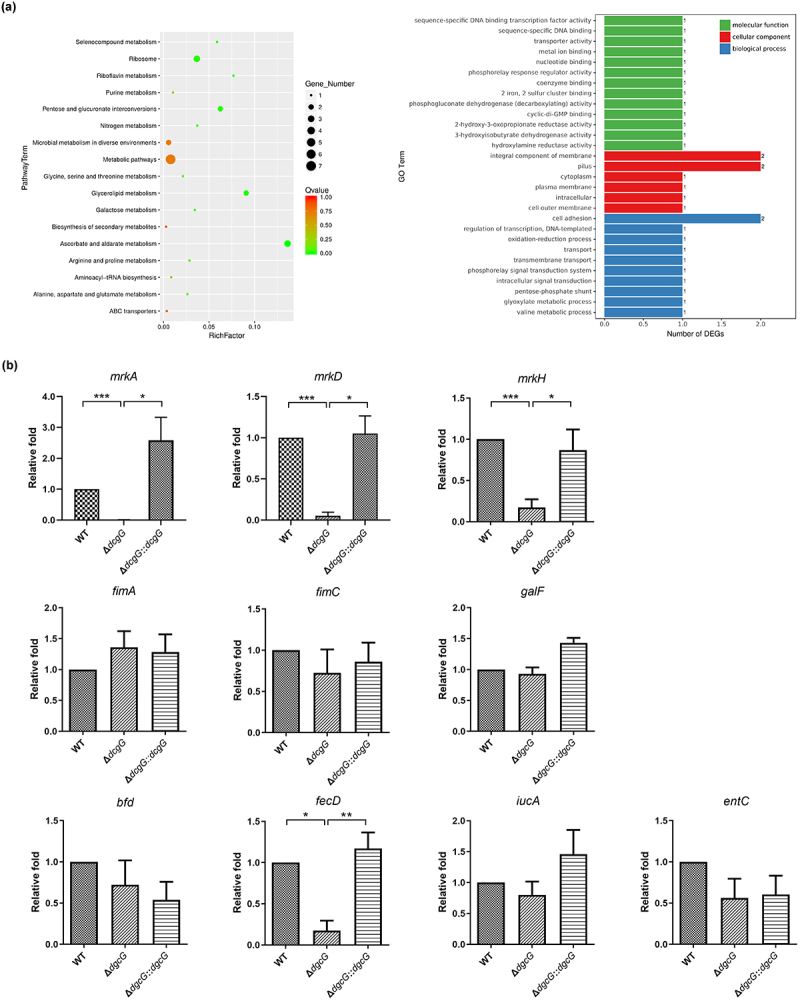
*K. pneumoniae* DgcG promoted expression of type 3 fimbrial and iron transport genes. (a) RNA-seq transcriptomic analysis to compare *K. pneumoniae* Ca0437 wild-type (WT) and Δ*dgcG* mutant. left graph: gene ontology (GO) categories showing differentially expressed genes between WT and Δ*dgcG* strains; right graph: venn diagram analysis of differentially expressed genes between WT and Δ*dgcG* strains. Data from two independent experiments. (b) RT-qPCR to assess potential effects on expression of specific gene (as indicated). *K. pneumoniae* Ca0437 wild-type (WT), *dgcG*-deletion mutant (Δ*dgcG*), and complementation strain (Δ*dgcG::dgcG*) were determined and compared. Data represent the mean and standard error of the mean from 3 independent experiments. *, *p* < 0.05; **, *p* < 0.01; ***, *p* < 0.001; analysis of variance followed by Bonferroni multiple comparisons test.

To dissect the underlying mechanisms, RNA-sequencing (RNA-seq) was conducted to compare the transcriptomes of WT and Δ*dgcG* strains, revealing that *dgcG* deletion affected genes involved in metabolism, transmembrane transport, cell adhesion, and iron acquisition (Figure 4a). Among the 5211 genes analyzed, expression of 47 genes was significantly altered, including 27 downregulated genes (log₂ fold change ranging from −0.49 to −2.87) and 20 upregulated genes (log₂ fold change ranging from 0.48 to 0.75). Twenty-nine of these genes showed expression changes greater than ~ 1.5-fold (log₂ fold change >0.58 or <−0.58), including 18 downregulated and 11 upregulated genes (Table 2). Notably, several type 3 fimbrial genes (*mrk*) and iron transport-related genes *bfd* and *fecD* were downregulated upon deletion of *dgcG*. The raw RNA-seq gene expression data are provided in Dataset 2 of the supplementary files.

**Table 2.** DgcG-regulated genes identified in RNA-seq transcriptomic analysis.

Gene ID	Predicted function (gene)^a^	Accession number	Fold change (log_2_)^*b*^
**Down**			
Gene468	Diguanylate cyclase (*dgcG*)	WP_002887653.1	−2.873678777
Gene4012	Type 3 fimbria adhesin subunit MrkD (*mrkD*)	WP_004900381.1	−1.700913247
Gene4008	Type 3 fimbria protein MrkH (*mrkH*)	WP_004152886.1	−1.176812053
Gene4013	Type 3 fimbria usher protein MrkC (*mrkC*)	WP_000813718.1	−1.131645233
Gene1475	Hypothetical protein	WP_002895851.1	−0.97164089
Gene4014	Molecular chaperone	WP_014599251.1	−0.835499923
Gene4009	LuxR family transcriptional regulator	WP_004149657.1	−0.801099084
Gene734	tRNA-Ala		−0.778722138
Gene4011	Type 3 fimbria minor subunit MrkF (*mrkF*)	WP_002916122.1	−0.732081349
Gene4493	Bacterioferritin-associated ferredoxin (*bfd*)	WP_004900993.1	−0.729630352
Gene4015	Type 3 fimbria major subunit MrkA (*mrkA*)	WP_002916128.1	−0.7220177
Gene2783	Iron ABC transporter permease (*fecD*)	WP_004148676.1	−0.698754303
Gene397	Hypothetical protein	WP_002887388.1	−0.664076289
Gene334	Hypothetical protein	WP_004192273.1	−0.617954192
Gene3138	tRNA-Ser		−0.60136311
Gene2858	D-amino acid dehydrogenase	WP_004180373.1	−0.601344184
Gene214	Primosomal replication protein N	AEJ96132.1	−0.592762114
Gene2022	Transcriptional regulator	AEJ97850.1	−0.584059681
**Up**			
Gene4265	Dihydroxyacetone kinase subunit DhaK (*dhaK*)	WP_002917682.1	0.752825101
Gene4319	Alpha-dehydro-beta-deoxy-D-glucarate aldolase	WP_004900848.1	0.751904498
Gene1587	Oxidoreductase	WP_004147771.1	0.684258867
Gene3875	Glucarate dehydratase	WP_002915223.1	0.669223088
Gene208	3-dehydro-L-gulonate-6-phosphate decarboxylase UlaD (*ulaU*)	WP_002885684.1	0.605177494
Gene378	Hypothetical protein	WP_002887282.1	0.600854841
Gene1588	Hydroxylamine reductase	AEJ97439.1	0.59937008
Gene1486	Putative periplasmic binding protein/LacI transcriptional regulator	AEJ97336.1	0.59760055
Gene4875	Permease	AEK00571.1	0.591763952^a^
Gene4266	Dihydroxyacetone kinase subunit DhaL(dhaL)^b^	AEJ99993.1	0.5904176
Gene4334	Protease	AEK00063.1	0.588401486

^a^Analysis using NCBI-BLAST.

^b^Twenty-nine genes with expression changes more than ~ 1.5 folds (log2 fold change > 0.58 or < −0.58), including 18 genes downregulated and 11 genes upregulated.

RT-qPCR was further preformed to examine the expression of individual fimbrial and iron-related genes (Figure 4b). We verified reduced expression of type 3 fimbrial genes *mrkA*, *mrkC*, *mrkH*, and iron transport gene *fecD* in Δ*dgcG*, with genetic complementation restoring the defects. For comparison, other genes relating to *K. pneumoniae* colonization and virulence were also determined (Figure 4b). Expression of type 1 fimbrial genes (*fimA* and *fimC*), siderophore genes (*iucA* and *entC*), and CPS gene *galF* were not significantly altered. These findings indicate that DgcG promotes the expression of genes relating to type 3 fimbriae production and iron transport.

### *K. pneumoniae* DgcG enhances in vivo virulence by gastrointestinal infection

**Figure 5.**
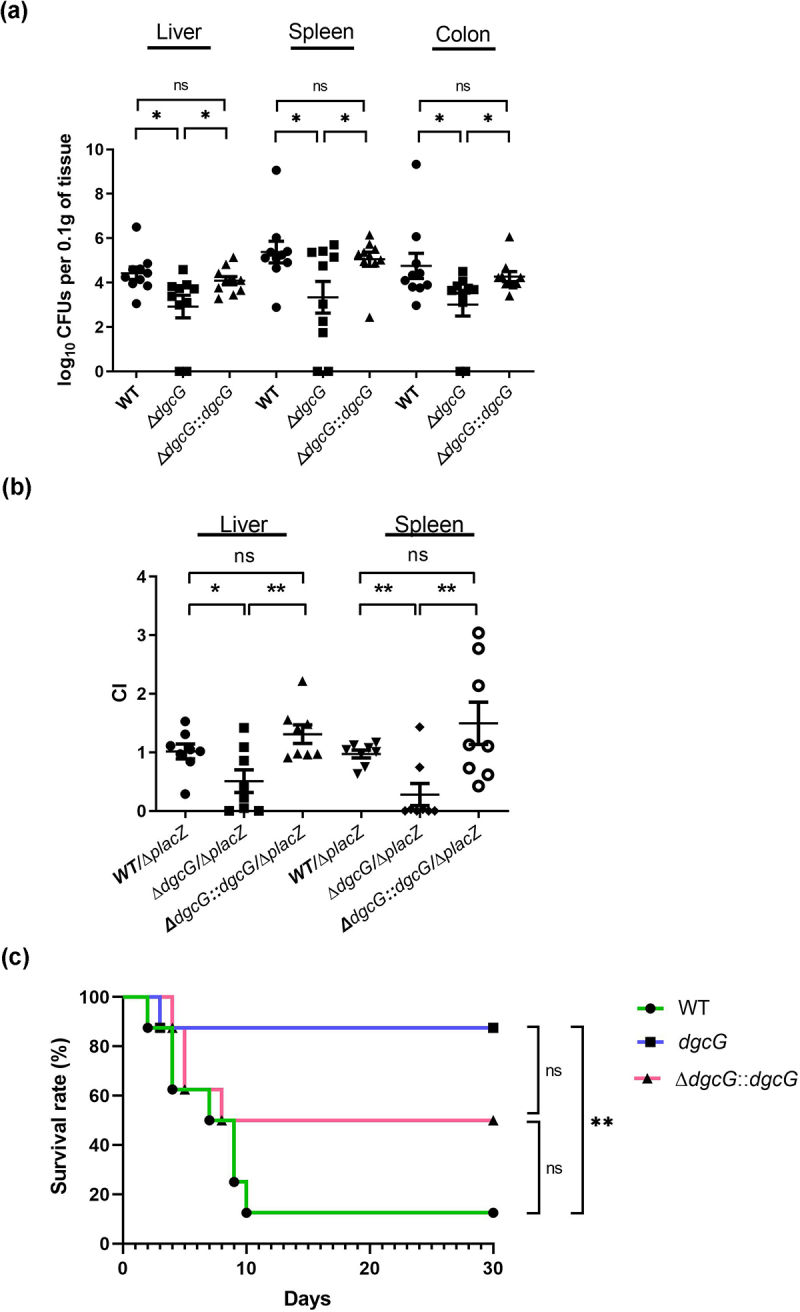
*K. pneumoniae* DgcG contributed to *in vivo* virulence in a gastrointestinal infection model. (a) Bacterial loads in mice. *K. pneumoniae* WT, Δ*dgcG* or Δ*dgcG::dgcG* was administered IG to each of four BALB/cByl mice (ten mice per group). Bacterial levels in liver, spleen, and colon were determined at 72 h post-infection. Bacterial numbers (expressed as log_10_ CFU) were standardized per 0.1 g of wet organ weight. *, *p* < 0.05; ns, not significant; analysis of variance followed by Bonferroni multiple comparisons test. Abbreviation: ns, not significant. (b) *in vivo* competition assays. Each test strain (WT, Δ*dgcG* or Δ*dgcG::dgcG*) was compared with the Δp*lacZ* mutant strain in BALB/cByl mice (eight mice per group) by IG inoculation. Following bacterial recovery on IPTG/X-Gal plates, the ratio of LacZ-positive (blue) to LacZ-negative (white) colonies in the liver or spleen of each mouse was determined. The competitive index (CI) was defined as (outputΔp*lacZ*/output_test strain_)/(inputΔp*lacZ*/input_test strain_). Each symbol represents CI for each inoculum, with the medians shown by bars. *, *p* < 0.05; **, *p* < 0.001; ns, not significant; Wilcoxon signed rank test. (c) Mouse survival analysis. BALB/cByl mice (eight mice per group) were infected intragastrically (IG) with *K. pneumoniae* Ca0437 wild type (WT) or isogenic Δ*dgcG* mutant. Survival of mice was monitored for 4 weeks. *, *p* < 0.05; **, *p* < 0.01; ns, not significant; log-rank test.

The impact of DgcG on *in vivo* virulence of *K. pneumoniae* was evaluated using a murine gastrointestinal infection model [30]. We observed that bacterial loads in the liver, spleen, and colon were significantly reduced in Δ*dgcG*-infected mice (Figure 5a). *In vivo* competition assays showed that Δ*dgcG* had a reduced competitive index compared to WT in both the liver and spleen, indicating that the attenuated ability of Δ*dgcG* to survive and spread in the host (Figure 5b). These results suggest that DgcG plays a critical role in promoting gastrointestinal colonization, bacterial survival, and dissemination within the host. By intragastrical inoculation, mice infected with Δ*dgcG* exhibited significantly attenuated virulence, with a 30-day survival rate of 87.5% (7/8) compared to 12.5% (1/8) in the wild-type (WT) group (*p* = 0.0062, log-rank test) (Figure 5c). This indicated that DgcG contributed to *in vivo* virulence of *K. pneumoniae* and influenced host mortality.

### High prevalence and conservation of dgcG in diverse *K. pneumoniae* isolates

The prevalence of *dgcG* in *K. pneumoniae* strains from clinical and gastrointestinal colonization sources was determined (Table 3). The overall prevalence of *dgcG* was 98.88% (177/179) in 179 tested strains, with 98.17% (107/109) in clinical isolates and 100% (70/70) in gut isolates. In clinical strains, lower prevalence rates were observed in wound (9/10, 90%) and CSF (16/17, 94.12%) isolates. The sequence types (STs) and capsule locus (KL) types of gut isolates were compared (supplementary Table S4). Despite the diversity in STs and KL types, all gut isolates carried *dgcG*. The amino acid sequence similarity of the putative DgcG ranged from 83.54% to 100%, with the majority of strains (67.1%, 47/70) possessing DgcG with 100% identity. These findings indicate a high prevalence and conservation of *dgcG* among genetically diverse *K. pneumoniae* gut isolates.

**Table 3.** Detection of *dgcG* in *K. pneumoniae* strains from different sources.

Sources	Number of test strains	Positive of dgcG	Prevalence (%)
**Gut colonization strains**	70	70	100.00
**Clinical strains**	109	107	98.17
Blood isolates	23	23	100.00
CSF isolates	17	16	94.12
Sputum isolates	29	29	100.00
Urine isolates	21	21	100.00
Wound isolates	10	9	90.00
Bile isolates	9	9	100.00
**Total**	179	177	98.88

Notes: PCR-based detection. Gut colonization strains were stool isolates from the subjects of health examination. Clinical strains were collected from different specimens of the patients as indicated.

The putative DgcG in 636 *K. pneumoniae* genomes from publicly available NCBI databases [40–42] was further analyzed (Table 4). The overall prevalence of the *dgcG* gene was 100%. The amino acid sequence similarity of the putative DgcG ranged from 95.98% to 100%, with 80.5% of strains (514/636) carrying DgcG with 100% identity. While a few strains encoded DgcG proteins of varying lengths, most strains (98.6%, 627/636) carried a 348-amino-acid DgcG. The presence or conservation of DgcG was not associated with hvKp, MDR-Kp, or specific ST or KL types. Notably, even within the same ST-KL group (e.g. ST23-KL1), variations in DgcG sequence similarity or protein length were observed. Conversely, DgcG with 100% identity was found across distinct ST-KL types. These results indicate again that *dgcG* is highly prevalent and conserved across genetically diverse *K. pneumoniae* clones. Table S5 showed putative DgcGs in the well-known reference strains. The putative DgcG was highly conserved among 9 *K. pneumoniae* reference genomes. Seven strains, including hypervirulent reference strains NTUH-K2044, CG43, SGH10 and multidrug-resistant strains HS11286 and NJST258_1, carried DGCs with 100% amino-acid sequence similarities to Ca0437 DgcG. The other two strains, MGH 78,578 and Kp342, harboured a putative DgcG with the sequence similarities of 99.63% and 95.98%, respectively. These findings suggest that *dgcG* is ubiquitous and conserved across *K. pneumoniae* strains. This gene likely encodes an essential and core DGC involved in the regulation of important physiological functions.

**Table 4.** Comparison of *dgcG* in *K. pneumoniae* genomes from the public available database.

ST^a^	KL^*b*^	N^*c*^	*dgcG*-positive N (prevalence rate)	DgcG length^*d*^ (N)	Amino acid sequence identity^*e*^ (N)
**HvKp**^f^**related**					
ST23	KL1	53	53 (100%)	348 a.a. (51)	100% (51)
				269 a.a. (2)	100% (2)
ST86	KL2	24	24 (100%)	348 a.a. (24)	100% (22), 99.71% (2)
ST65	KL2	6	6 (100%)	348 a.a. (6)	100% (6)
ST29	KL54	6	6 (100%)	348 a.a. (4)	99.71% (3), 100% (1)
				347 a.a. (2)	99.71% (2)
ST412	KL57	4	4 (100%)	348 a.a. (4)	100% (4)
ST218	KL57	3	3 (100%)	348 a.a. (3)	100% (2), 99.71% (1)
**MDR-Kp**^g^**related**					
ST11	KL24	9	9 (100%)	348 a.a. (9)	100% (9)
	KL47	9	9 (100%)	348 a.a. (9)	100% (9)
	KL64	5	5 (100%)	348 a.a. (5)	100% (5)
	KL105	3	3 (100%)	348 a.a. (3)	100% (3)
	KL125	2	2 (100%)	348 a.a. (2)	100% (2)
	KL103	1	1 (100%)	348 a.a. (1)	100% (1)
ST15	KL24	9	9 (100%)	348 a.a. (9)	100% (9)
	KL112	8	8 (100%)	348 a.a. (7) 180 a.a. (1)	100% (7) 99.44% (1)
	KL19	6	6 (100%)	348 a.a. (6)	100% (6)
	KL39	2	2 (100%)	348 a.a. (2)	100% (2)
	KL28	1	1 (100%)	348 a.a. (1)	100% (1)
ST258	KL107	14	14 (100%)	348 a.a. (13) 269 a.a. (1)	100% (13) 99.71% (1)
	KL106	10	10 (100%)	348 a.a. (10)	100% (10)
	KL74	2	2 (100%)	348 a.a. (2)	100% (2)
	KL63	1	1 (100%)	348 a.a. (1)	100% (1)
ST512	KL107	13	13 (100%)	348 a.a. (13)	100% (13)
ST307	KL102	12	12 (100%)	348 a.a. (12)	100% (12)
ST147	KL64	11	11 (100%)	348 a.a. (11)	99.43% (10), 100% (1)
	KL10	2	2 (100%)	348 a.a. (2)	99.43% (2)
	KL20	1	1 (100%)	348 a.a. (1)	99.43% (1)
	KL111	1	1 (100%)	348 a.a. (1)	99.43% (1)
	KL125	1	1 (100%)	348 a.a. (1)	99.42% (1)
ST101	KL17	11	11 (100%)	348 a.a. (11)	100% (11)
	KL106	2	2 (100%)	348 a.a. (2)	100% (2)
	KL62	1	1 (100%)	348 a.a. (1)	100% (1)
	KL64	1	1 (100%)	348 a.a. (1)	99.43% (1)
ST14	KL2	10	10 (100%)	348 a.a. (10)	100% (8), 99.71 (2)
	KL16	3	3 (100%)	348 a.a. (3)	100% (3)
	KL64	1	1 (100%)	348 a.a. (1)	99.71% (1)
ST48	KL62	6	6 (100%)	348 a.a. (6)	100% (6)
ST111	KL63	4	4 (100%)	348 a.a. (4)	99.71% (4)
ST29	KL30	2	2 (100%)	348 a.a. (2)	100% (2)
**Other dominant type**					
ST20	KL28	7	7 (100%)	348 a.a. (7)	100% (7)
ST45	KL24	6	6 (100%)	348 a.a. (6)	100% (6)
ST16	KL51	6	6 (100%)	348 a.a. (6)	100% (6)
ST231	KL51	6	6 (100%)	348 a.a. (6)	100% (6)
ST340	KL15	6	6 (100%)	348 a.a. (6)	100% (6)
ST17	KL25	5	5 (100%)	348 a.a. (5)	100% (5)
ST268	KL20	5	5 (100%)	348 a.a. (5)	100% (5)
ST336	KL25	5	5 (100%)	348 a.a. (5)	100% (5)
ST25	KL2	4	4 (100%)	348 a.a. (4)	99.71% (4)
ST35	KL22	4	4 (100%)	348 a.a. (4)	100% (4)
ST299	KL7	4	4 (100%)	348 a.a. (4)	99.71% (4)
ST437	KL36	4	4 (100%)	348 a.a. (4)	100% (4)
**Other strains with different length of DgcG**					
ST1754	KL62	1	1 (100%)	234 a.a. (1)	99.57% (1)
ST321	KL3	1	1 (100%)	269 a.a. (1)	99.63% (1)
ST660	KL16	1	1 (100%)	288 a.a. (1)	99.65% (1)
ST1741	KL104	1	1 (100%)	391 a.a. (1)	98.56% (1)
**The others**		310	310 (100%)	348 a.a. (310)	100% (233), 99.71% (65), 99.43% (9), 99.72% (1), 97.70% (1), 95.98% (1)
**Total**		**636**	**636 (100%)**	**348 a.a. (626)**	**95.98% − 100%**
				**269 a.a. (4)**	**99.63% − 100%**
				**347 a.a. (2)**	**99.71%**
				**180 a.a. (1)**	**99.44%**
				**234 a.a. (1)**	**99.57%**
				**288 a.a. (1)**	**99.65%**
				**391 a.a. (1)**	**98.56%**

^a^ST: sequence type.

^b^KL: K locus type.

^c^N: number of strains.

^d^a.a.: amino acid number.

^e^Sequence identity determined using Clustal Omega (https://www.ebi.ac.uk/Tools/msa/clustalo/), compared with Ca0437 DgcG (348 a.a.).

^f^HvKp: hypervirulent K. pneumoniae.

^g^MDR-Kp: multidrug-resistant K. pneumoniae.

## Discussion

C-di-GMP, though absent in higher eukaryotes, is ubiquitous in bacteria where it regulates various cellular processes. DGCs have been identified as promising drug targets for developing therapeutics that interfere with the second messenger-signaling networks in pathogenic bacteria [14–17]. Most bacteria possess multiple DGCs and PDEs to respond to diverse environmental signals and regulate a variety of cellular functions [18,19]. The multiplicity of these enzymes, along with their structural diversity, raises questions about the specificity and complexity of c-di-GMP regulatory networks. In this study, we identified an essential DGC, DgcG, playing a critical role in *K. pneumoniae* pathogenesis. This *K. pneumoniae* DGC regulates cell interactions, biofilm formation, and iron utilization. Based on the study of a bacteremia and liver abscess-inducing strain Ca0437, we demonstrated the multiple functions of DgcG in controlling virulence-related traits in *K. pneumoniae*. Given the global threat posed by MDR and hypervirulent *K. pneumoniae*, DgcG could be a promising target for developing new therapeutic agents. For example, small-molecule libraries can be screened for drug discovery to identify DGC inhibitors as the novel antimicrobials.

Our findings expand the understanding of c-di-GMP signaling in *K. pneumoniae*-host interactions. Adherence to host cells and subsequent tissue colonization is often a crucial first step in establishing infection. Deletion of *dgcG* led to significant reductions in *K. pneumoniae* adherence to both intestinal (Caco-2) and bladder (T24) epithelial cells, suggesting that DgcG contributes to epithelial colonization in both gastrointestinal and urinary tracts. These results are consistent with previous studies highlighting the role of c-di-GMP signaling in modulating fimbrial expression and host cell attachment in various Gram-negative pathogens [21,43–45]. We found that DgcG did not significantly affect adherence to retinal pigment epithelial cells and macrophages. Our previous study also demonstrated no impact on adherence to A549 lung epithelial cells and HepG2 hepatocytes [30]. Therefore, DgcG seems to promote *K. pneumoniae* interactions with specific cell types, highlighting the importance of this DGC in selective tissue colonization. Notably, RNA-seq and RT-qPCR data showed that DgcG regulated type 3 fimbriae but not type 1 fimbriae, indicating a pathway-specific regulation mechanism. In *K. pneumoniae*, type 1 fimbriae (encoded by the *fim* operon) was reported essential for UTI initiation [46], while type 3 fimbriae (encoded by the *mrk* operon) promoted biofilm formation and binding to collagen and abiotic surfaces [47,48]. In agreement with our findings, previous research has shown that MrkH, a PilZ-domain transcriptional activator, regulated *mrk* expression in response to c-di-GMP [21]. A potential regulatory pathway could be that DgcG influences cell adherence through the c-di-GMP-dependent protein MrkH, which further regulates *mrk* operon and therefore type 3 fimbrial expression.

Biofilm formation, another critical virulence factor regulated by c-di-GMP, was impaired in the absence of DgcG. This phenotype was validated by both crystal violet staining and SEM imaging. Several studies have linked intracellular c-di-GMP levels to enhanced biofilm formation in *K. pneumoniae* and other enteric pathogens [21,23,49–52]. In *K. pneumoniae*, type 3 fimbriae has been considered as the primary factor to influence biofilm formation under c-di-GMP control [21,53]. On the other hand, we did not observe changes in capsule production or mucoviscosity upon *dgcG* deletion, in agreement with studies showing that capsule biosynthesis in *K. pneumoniae* may be more closely linked to RmpA, RcsAB, or other capsule regulators [54]. Consistent with our findings, a study of a *K. pneumoniae* UTI strain (AJ218) reported a distinct *K. pneumoniae* diguanylate cyclase, YfiN (DgcN), upregulating biofilm formation and type 3 fimbriae expression [21]. Sequence alignment between DgcG and YfiN with a similarity of only 21.30%. This indicates that *K. pneumoniae* employs multiple diverse DGCs to regulate similar functions, such as biofilm formation and expression of fimbriae, potentially allowing the bacterium to finely tune these critical processes in response to varying environmental signals.

DgcG played a role in iron acquisition mainly in the growth phase, as Δ*dgcG* mutants were deficient in utilizing Fe^3 +^ or Fe^2 +^ between 0 and 8 hours but not by 24 hours. Lack of *dgcG* caused the lower efficiency and the delayed kinetics to resume growth in the presence of iron by the iron-starved strain. Some possible reasons could contribute to the difference between the 8-hour and 24-hour time points. For example, DgcG may regulate the timely activation of iron acquisition systems or be involved in growth phase-dependent regulation [55]. There could also be compensatory mechanisms such as activation of alternative iron uptake pathways [56]. Our data indicate that while DgcG might not be essential for iron uptake, it enhances the rate and efficiency of iron utilization during periods of rapid growth. RNA-seq results during the log phase revealed downregulation of iron transport genes such as *fecD* and *bfd*, supporting the idea that c-di-GMP signaling modulates the expression of genes involved in iron metabolism. Iron is essential for *K. pneumoniae* survival and pathogenicity, and iron uptake systems are tightly regulated in response to host-imposed nutritional immunity. To our knowledge, this is the first report of a DGC regulating iron metabolism in *K. pneumoniae*. Studies in *Pseudomonas aeruginosa* demonstrated that c-di-GMP modulates siderophore production and iron acquisition in response to environmental signals [57]. Our data suggest a similar role for c-di-GMP in fine-tuning iron metabolism in *K. pneumoniae*, although further studies are needed to define the mechanistic basis of this regulation.

The *in vivo* mouse model of gastrointestinal infection further validated the role of DgcG in virulence of *K. pneumoniae*. To our knowledge, this is the first study to demonstrate the contributions of a DGC and c-di-GMP signaling to *K. pneumoniae* GI infection. The Δ*dgcG* mutant exhibited significantly reduced gastrointestinal colonization and attenuated virulence to affect host mortality, underscoring the importance of intestinal interactions in *K. pneumoniae* pathogenesis. Bacterial burden and *in vivo* competition analyses indicated that DgcG was important for bacterial survival and spread within the host from the gastrointestinal tract to cause systemic infection. Our data suggest that DgcG functions as an upstream regulator of both type 3 fimbriae and iron acquisition, two key traits implicated in gastrointestinal colonization and systemic dissemination [12,58,59]. By study of a bacteremia and liver abscess-inducing strain (Ca0437), we revealed c-di-GMP involvement in GI interaction and systemic infection, contributing to the knowledge about the role of c-di-GMP signaling in *K. pneumoniae* pathogenesis. The c-di-GMP signaling has been reported to regulate *Salmonella* gastrointestinal colonization and *in vivo* virulence [60]. In *K. pneumoniae*, previous studies about c-di-GMP in pathogenesis mainly focused on respiratory infections [61,62]. One study demonstrated that increased c-di-GMP levels by expression of a heterologous DGC in a cystitis isolate (TOP52) attenuated bacterial virulence in a murine pneumonia model, likely through enhanced type 1 fimbrial expression [61]. Another study showed that local intranasal (i.n.) or systemic subcutaneous (s.c.) administration of synthetic c-di-GMP in mice induced protective immunity against lung infections by *K. pneumoniae* [62]. The roles of *K. pneumoniae* DgcG in distinct types of infections warrant further exploration.

The high prevalence and conservation of *dgcG* among gut-derived and clinical *K. pneumoniae* isolates, including hypervirulent and multidrug-resistant lineages, suggest that DgcG encodes a core signaling enzyme that is under strong evolutionary conservation. The presence of highly conserved DgcG in diverse ST and KL types, and its absence of linkage to specific lineages, highlight its potential as a broad-spectrum target for therapeutic development.

In conclusion, this study characterized a novel *K. pneumoniae* DGC and demonstrated its contributions to gastrointestinal interactions and pathogenesis. DgcG functions as a diguanylate cyclase that promotes to intestinal adherence, biofilm formation, and iron metabolism. Given its influence on bacterial virulence and conservation among *K. pneumoniae* isolates, DgcG represents a potential therapeutic target, especially in the context of the growing antimicrobial resistance crisis. Our findings provide new insights into the c-di-GMP-mediated regulation of *K. pneumoniae* pathogenicity, particularly in its interactions with the host GI tract. Future research should investigate the potential stimuli and downstream effectors of DgcG involved in c-di-GMP signaling networks that regulate bacterial virulence.

## Supplementary Material

Dataset 2_RNA seq raw data.xlsx

Dataset 1_gut strains and public genomes.xlsx

Clean Copy of Supplementary Methods and Materials- QVIR-2025-0134.R1.docx

## Data Availability

The data that support the findings of this study are available in this article, supplementary files, and Figshare (https://figshare.com) with DOI: https://doi.org/10.6084/m9.figshare.28517942. The sequencing data of *K. pneumoniae* Ca0437 *dgcG* is available through NCBI GenBank with the accession number PQ435165.
