# Supplementary material for: A novel Klebsiella pneumoniae diguanylate cyclase contributes to intestinal cell adhesion, biofilm formation, iron utilization, and in vivo virulence by gastrointestinal infection
Source: Virulence. 2025 Aug 6;16(1):2544882. doi: 10.1080/21505594.2025.2544882 (PMC12355678; doi:10.1080/21505594.2025.2544882)
Supplement: Clean Copy of Supplementary Methods and Materials- QVIR-2025-0134.R1.docx [file KVIR_A_2544882_SM4944.docx]

**Supplemental Materials**

**Supplementary Materials and Methods**

**Cell adherence assays**

Four different cell lines were tested in adherence assays. Caco-2 human intestinal epithelial cells were grown in Dulbecco’s modified Eagle’s medium (DMEM) supplemented with 10% heat-inactivated fetal bovine serum (FBS) and 1% nonessential amino acids (Gibco). T24 human urinary bladder epithelial cells were grown in McCoy’s 5a medium supplemented with 10% FBS. ARPE-19 human retinal pigment epithelial cells were grown in Dulbecco’s Modified Eagle Medium Nutrient Mixture F-12 (DMEM/F-12) supplemented with 10% FBS. RAW 264.7 murine macrophages were grown in RPMI 1640 medium supplemented with 10% FBS. The adherence assays were performed as described previously [2]. Cells (~5×10^5^ cells per well) were seeded in 24-well plates. Mid-log-phase *K. pneumoniae* resuspended in FBS-free media were added to cells at a multiplicity of infection (MOI) of 50. After centrifugation at 200×g for 5 min, plates were incubated for 20 min in a humidified 5% CO2 atmosphere at 37°C (or 4°C for macrophages) [4], followed by three times of wash with phosphate-buffered saline (PBS). The bacteria then were released by 0.2% Triton X-100 (Sigma-Aldrich) and recovered on LB plates for quantification of colony-forming units (CFU). The adherence rate was the proportion of the inoculum that adhered.

**Expression of *dgcG* and quantification of c-di-GMP using high performance liquid chromatography (HPLC)**

To verify gene function, *dgcG* from *K. pneumoniae* Ca0437 was cloned and expressed in *E. coli* BL21 (DE3). *K. pneumoniae dgcG* was PCR-amplified and cloned into pJET1.2 vector using CloneJET PCR Cloning Kit (Thermo Scientific™, #K1231), and further expressed in *E. coli* BL21 (DE3) under isopropylthio-β-galactoside (IPTG). *E. coli* BL21 (DE3) transformed by pJET1.2 vector or by pJET1.2::*dgcG* were treated with 0.4mM Isopropyl β-D-1-thiogalactopyranoside (IPTG) for induction or ddH2O for 4h-incubation at 37°C. Bacterial cells were further harvested and cellular c-di-GMP was extracted and quantified.

For c-di-GMP extraction, bacteria were pelleted and resuspended in ice-cold extraction solvent of acetonitrile/methanol/water (40/40/20, v/v/v) for 15 min incubation on ice, followed by 95 °C heating for 10 min. After cooling, cell suspension was centrifuged at 4 °C (20000×g, 15 min). The supernatants (containing nucleotides) were collected and then dried under vacuum by centrifugal evaporation until dry pellets were obtained.

For c-di-GMP detection and quantification, the dry pellets were resuspended in water for following HPLC analysis according to previously described methods [5]. The HPLC analysis was conducted on a Waters Alliance e2695 LC system equipped with a Waters 2998 photodiode array detector. The chromatographic separation was performed on an Agilent Zorbax XDB-C18 column (3.0 mm I.D. x 150 mm, 3.5 µm, 80 Å). The mobile phase consisted of water (mobile phase A) and acetonitrile (mobile phase B). A linear gradient elution over 30 min was employed at a flow rate of 0.7 mL/min, starting with 0% buffer B at 0-5 min, increasing to 20% buffer B at 5-10 min, reaching 30% at 10-15min, escalating to 90% at 15-21 min, and returning to 0 % buffer B by 22 min. For quantification, a c-di-GMP standard curve was generated using 1, 5, 10, 15, 20, 25 ppm c-di-GMP (Sigma-Aldrich).

**String test, low-speed centrifugation, and quantification of capsular polysaccharide (CPS)**

String test was performed to determine hypermucoviscosity (HMV) of *K. pneumoniae*. HMV phenotype was defined by formation of viscous strings >5 mm long [6]. To determine the levels of mucoviscosity, a low-speed centrifugation test was performed. Briefly, equal numbers of exponential phase-cultured bacteria were centrifuged at 1000 g for 5 min. The supernatant was subjected to measurement of the absorbance at 600 nm. To quantify capsular production, CPS of *K. pneumoniae* Ca0437 strain, which is belonged to K2 capsular type, were extracted and the levels were determined according to previously described methods [7]. Briefly, 500 μL of overnight grown bacteria was mixed with 100 μL of 1% Zwittergent 3-14 (Sigma-Aldrich, Milwaukee, WI) in 100 mM citric acid (pH 2.0) and then incubated at 50°C for 20 min. After centrifugation, 250 μL of the supernatants containing CPS were precipitated by 1 mL of absolute ethanol. The pellets were dried and dissolved in 200 μL of distilled water, followed by addition of 1,200 μL of 12.5 mM borax in H_2_SO4. After vigorously vortex, the mixtures were boiled for 5 min, cooled, and added by 20 μL of 0.15% 3-hydroxydiphenol (Sigma-Aldrich). The absorbance at 520 nm were measured. The uronic acid contents in K2 CPS were determined from a standard curve of glucuronic acid and expressed as micrograms per 10^9^ CFU.

**Mouse husbandry** **for experiments**

All animal experiments were approved by the Institutional Animal Care and Use Committee (IACUC) of National Taiwan Ocean University (NTOU) (IACUC-109060) and adhered to Taiwan’s Animal Protection Act. The study has adhered to the ARRIVE guidelines. Mouse survival, bacterial load, and *in vivo* competition assays were performed in a murine gastrointestinal infection model using five-week-old female BALB/cByl mice purchased from the National Laboratory Animal Center, Taiwan (<https://www.nlac.narl.org.tw/>). A total of 24 mice were used for survival analysis, 30 mice for bacterial load analysis, and 24 mice for *in vivo* competition assays. Sample size was decided based previous studies [2,8]. During the experiments, all animals were housed in SPF-grade animal facilities within a specific pathogen-free environment. The housing conditions included a 12-hour light/dark cycle, with controlled ambient temperature and humidity levels. From entry into the facility, all mice were housed under Animal Biosafety Level 2 (ABSL-2) conditions, where they acclimated to the facility for at least 6 days. Prior to the start of the study, all mice were monitored daily during routine animal health check; healthy individuals were included in the study. Mice were randomly allocated in cages and divided into groups. For mouse survival and *in vivo* competition assays, 8 mice per group were housed in two cages (4 mice per cage). For bacterial load assays, 10 mice per group were housed in two cages (5 mice per cage). Each group was randomly assigned to bacterial strains to test. Animal care staff were blinded to treatment to allow consistent care across all animals. The animal experiments complied with the requirements of the Institutional Animal Care and Use Committee of NTOU. Animals were euthanized using CO₂ inhalation after observation period ended.

**Supplementary References**

1. Hsu, C.R., et al., *The role of Klebsiella pneumoniae rmpA in capsular polysaccharide synthesis and virulence revisited.* Microbiology (Reading), 2011. **157**(Pt 12): p. 3446-3457.

2. Hsu, C.R., et al., *A Novel Role for the Klebsiella pneumoniae Sap (Sensitivity to Antimicrobial Peptides) Transporter in Intestinal Cell Interactions, Innate Immune Responses, Liver Abscess, and Virulence.* J Infect Dis, 2019. **219**(8): p. 1294-1306.

3. Huang, S.H., et al., *Role of the small RNA RyhB in the Fur regulon in mediating the capsular polysaccharide biosynthesis and iron acquisition systems in Klebsiella pneumoniae.* BMC Microbiol, 2012. **12**: p. 148.

4. Ares, M.A., et al., *H-NS Nucleoid Protein Controls Virulence Features of Klebsiella pneumoniae by Regulating the Expression of Type 3 Pili and the Capsule Polysaccharide.* Front Cell Infect Microbiol, 2016. **6**: p. 13.

5. Petrova, O.E. and K. Sauer, *High-Performance Liquid Chromatography (HPLC)-Based Detection and Quantitation of Cellular c-di-GMP.* Methods Mol Biol, 2017. **1657**: p. 33-43.

6. Fang, C.T., et al., *A novel virulence gene in Klebsiella pneumoniae strains causing primary liver abscess and septic metastatic complications.* J Exp Med, 2004. **199**(5): p. 697-705.

7. Lai, Y.C., H.L. Peng, and H.Y. Chang, *RmpA2, an activator of capsule biosynthesis in Klebsiella pneumoniae CG43, regulates K2 cps gene expression at the transcriptional level.* J Bacteriol, 2003. **185**(3): p. 788-800.

8. Hsieh, P.F., et al., *CadC regulates cad and tdc operons in response to gastrointestinal stresses and enhances intestinal colonization of Klebsiella pneumoniae.* J Infect Dis, 2010. **202**(1): p. 52-64.

**Legends for Datasets**

**Dataset 1.** The gut isolates and genomes from the public database used to analyze *dgcG* in this study. The first worksheet showing 70 gut isolates and the second worksheet showing 636 genomes from the public database. Sequence types (STs) and capsule locus types (KL types) were determined using Kleborate program. DgcG was identified using tBLASTn program and amino acid (a.a.) sequence identity was determined using Clustal Omega.

**Dataset 2.** The raw data of RNA-seq gene expression. The first worksheet showing 47 genes significantly altered, including 27 downregulated genes and 20 upregulated genes. The second worksheet showing all gene expression analyzed in RNA-seq.

**Supplementary Data**

**Table S1.** Bacterial strains used in this study.

| **Name of strains or plasmids** | **Description** | **Reference or source** |
| --- | --- | --- |
| *K. pneumoniae* |  |  |
| Ca0437 Wild type (WT) | Blood isolate from the patient with septicemia; K2 capsular type | [1] |
| Ca0437Δ*dgcG* | Unmarked genetic deletion of *dgcG* | [2] |
| Ca0437Δ*dgcG*::*dgcG* | Chromosomal genetic complementation of *dgcG* in Δ*dgcG* strain | This study |
| Ca0437Δp*lacZ* | Unmarked genetic deletion of *lacZ* promoter; for *in vivo* competition assays (see Methods) | [2] |
| EDAH clinical strains | 109 isolates from the patients: 23 blood isolates, 17 CSF isolates, 29 sputum isolates, 21 urine isolates, 10 wound isolates, and 9 bile isolates | This study |
| EDAH colonization strains | 70 isolates from the stool of the subjects of health examination | This study |
|  |  |  |
| *E. coli* |  |  |
| DH10B | For gene cloning | Thermo Scientific™ |
| BL21 (DE3) | For gene expression | Thermo Scientific™ |
|  |  |  |

Notes: EDAH, E-Da Hospital.

**Table S2.** PCR and RT-qPCR primers used in this study.

| **Gene** | **Sequences** | **Reference** |
| --- | --- | --- |
| PCR |  |  |
| *dgcG* | F: gag ggg gaa caa tgg ttg cg | This study |
|  | R: ggt cat aga ccc gct gac gta tac |  |
|  |  |  |
| RT-qPCR |  |  |
| *fimA* | F: tcg atc aaa ccg ttc agt tag g | [2] |
|  | R: acc gta gtg tcg caa tca tc |  |
| *fimC* | F: tgt gag aaa aac ggc aac ga | [2] |
|  | R: ctg gcg gcg aaa agc a |  |
| *mrkA* | F: tgc gaa cgt tta cct gtc tc | [2] |
|  | R: tca tcc tgt tta gtg cca tca g |  |
| *mrkD* | F: tcg tct atc ccg acg tct tt | [2] |
|  | R: cca gtc gta gga ggt gta ctt a |  |
| *mrkH* | F: cgt gga ctt tgc cga gtt tc | This study |
|  | R: cgg ata cct tta tcg aca ccg |  |
| *galF* | F: tgc gtc acc aga aca atc tc | [2] |
|  | R: cca caa agg caa ttc caa agg |  |
| *bfd* | F: ccg gca tgg tgg tta att cat c | This study |
|  | R: gtt tgt ttg tgc aat ggc gtc |  |
| *fecD* | F: caa ggg tat cgg tcg cca g | This study |
|  | R: cat cat cct gtt ctg tac cgc c |  |
| *iucA* | F: tct ccc ggc tta ttg ttg ata | [3] |
|  | R: gga agg ttt cgc aac tgg t |  |
| *entC* | F: acc ccg cca gct tta act t | [3] |
|  | R: tgt cct tct tta cgc agc ag |  |

**Table S3**. HPLC quantification of c-di-GMP concentrations.

| **Construct** | **IPTG induction** | **C-di-GMP concentration (ppm)** |
| --- | --- | --- |
| pJET vector | + | 9.52 |
|  | - | 7.44 |
|  |  |  |
| pJET::*dgcG* | + | 50.08 |
|  | - | 8.192 |

Notes: *E. coli* BL21 (DE3) was transformed with pJET1.2 vector only (pJET) or pJET1.2 harboring *dgcG* (pJET::*dgcG*). Gene expression was induced by 0.4mM IPTG for 4 h. Intracellular c-di-GMP was extracted and quantified using HPLC with c-di-GMP standards (see Supplementary Methods).

**Table S4.** Prevalence and similarity of *dgcG* in human gut isolates.

| **ST***^a^* | **KL***^b^* | **N***^c^* | ***dgcG*-positive N (prevalence rate)** | **DgcG length***^d^* **(N)** | **Amino acid sequence identity***^e^* **(N)** |
| --- | --- | --- | --- | --- | --- |
| **HvKp***^f^* **related** | |  |  |  |  |
| ST23 | KL1 | 1 | 1 (100%) | 348 a.a. (1) | 100% (1) |
| ST65 | KL2 | 2 | 2 (100%) | 348 a.a. (2) | 100% (2) |
| ST14 | KL2 | 1 | 1 (100%) | 348 a.a. (1) | 100% (1) |
| ST29 | KL54 | 1 | 1 (100%) | 348 a.a. (1) | 100% (1) |
|  |  |  |  |  |  |
| **Other dominant type** | |  |  |  |  |
| ST107 | KL142 | 2 | 2(100%) | 348 a.a. (1)  285 a.a. (1) | 95.98% (1)  100% (1) |
| ST299 | KL7 | 2 | 2 (100%) | 348 a.a. (2) | 100% (2) |
| ST643 | KL105 | 2 | 2 (100%) | 348 a.a. (2) | 100% (2) |
|  |  |  |  |  |  |
| **Others** |  |  |  |  |  |
| ST200 | KL111 | 1 | 1 (100%) | 237 a.a. (1) | 83.54% (1) |
| ST35 | KL143 | 1 | 1 (100%) | 100 a.a. (1) | 100% (1) |
| Other single ST-KL*^g^* | | 57 | 57 (100%) | 348 a.a. (57) | 100% (36), 99.71% (11), 99.43% (2), 97.7% (6), 97.41% (2) |
|  |  |  |  |  |  |
| **Total** |  | **70** | 1. **(100%)** |  |  |

Notes:

*a.* ST: sequence type

*b.* KL: K locus type

*c.* N: number of strains

*d.* a.a.: amino acid number

*e.* Sequence identity determined using Clustal Omega (<https://www.ebi.ac.uk/Tools/msa/clustalo/>), compared with Ca0437 DgcG (348 a.a.).

*f.* HvKp: hypervirulent *K. pneumoniae*

*g.* The other ST-KL type with only one strain (N=1). No common hvKp-related or MDR-Kp related types.

**Table S5.** Putative DgcG in the genomes of well-known *K. pneumoniae* reference strains.

| **Strain** | **Strain type*^a^*** | **ST*^b^*** | **KL*^c^*** | **Gene locus** | **Accession number** | **Amino acid number** | **Amino acid sequence identity (%)*^d^*** |
| --- | --- | --- | --- | --- | --- | --- | --- |
| NTUH-K2044 | Clinical isolate; hvKp | ST23 | KL1 | KP1_0782 | WP_141438639.1 | 348 | 100 |
| CG43 | Clinical isolate; hvKp | ST86 | KL2 | D364_RS25295 | WP_002887653.1 | 348 | 100 |
| SGH10 | Clinical isolate; hvKp | ST23 | KL1 | SGH10_RS23120 | WP_002887653.1 | 348 | 100 |
| MGH 78578 | Clinical isolate; MDR-Kp | ST38 | KL52 | KPN_04822 | ABR80168.1 | 269 | 99.63 |
| HS11286 | Clinical isolate; MDR-Kp (CRKP) | ST11 | KL103 | KPHS_06770 | AEW59375.1 | 348 | 100 |
| NJST258_1 | Clinical isolate; MDR-Kp (CRKP) | ST258 | KL107 | KPNJ1_04781 | AHM87183 | 348 | 100 |
| ATCC 43816 | Clinical pneumonia isolate | ST493 | KL2 | IT767_16020 | QPW32438.1 | 348 | 100 |
| Kp342 | Plant endophyte | ST146 | KL30 | KPK_RS22715 | WP_008807534.1 | 348 | 95.98 |
| KCTC2242 | 2,3-butanediol-producing Kp | ST375 | KL2 | KPN2242_02265 | AEJ96373.1 | 348 | 100 |

Notes:

*a.* HvKp: hypervirulent *K. pneumoniae*; MDR-Kp: multidrug-resistant *K. pneumoniae*; CRKP: carbapenemase-producing *K. pneumoniae*.

*b.* ST: sequence type.

*c.* KL: K locus type.

*d*. compared with Ca0437 DgcG (348 amino acid) by Clustal Omega (<https://www.ebi.ac.uk/Tools/msa/clustalo/>).
